# Incidence, risk factors and outcomes of BCGosis following BCG vaccination in infants: a systematic review and meta-analyses

**DOI:** 10.3389/fimmu.2025.1615039

**Published:** 2025-12-11

**Authors:** Fathima Raahima Riyas Mohamed, Mudasar Nisar, Ismail Saeed, Mohammed Rushdhi Irfan, Ahlaam Khalid, Muneeb Faiz, Ahmed F. Younis, Shahan Javed, Arya Sen, Asma Azam, Muhabat Adeola Raji, Abrar Barakzai, Atef Mohamed Shibl, Garwin Kim Sing

**Affiliations:** 1 College of Medicine, Alfaisal University, Riyadh, Saudi Arabia; 2 Services Institute of Medical Sciences, Lahore, Pakistan; 3 Gomal Medical College, Dera Ismail Khan, Pakistan; 4 King Edward Medical University, Lahore, Pakistan; 5 Damietta Faculty of Medicine, Al-Azhar University, Cairo, Egypt; 6 Kolkata Medical College, Kolkata, West Bengal, India; 7 Karachi Medical and Dental College (KMDC), Karachi, Pakistan; 8 Department of Microbiology/Immunology, College of Medicine, Alfaisal University, Riyadh, Saudi Arabia; 9 Department of Pathology, College of Medicine, Alfaisal University, Riyadh, Saudi Arabia

**Keywords:** Bcgosis, BCG vaccine, immunodeficiency, tuberculosis, neonatal vaccination

## Abstract

**Introduction:**

The Bacille Calmette-Guerin (BCG) vaccine is widely administered in countries with high tuberculosis (TB) prevalence to protect against severe forms of childhood TB. Despite its efficacy, the vaccine can lead to adverse effects like BCGosis, a severe but rare condition marked by systemic granulomatous inflammation. This is because the vaccine is comprised of attenuated BCG bacteria which has the potential to cause uncontrolled dissemination beyond the injection site. Immunocompromised children are particularly vulnerable to this side effect. Through a systematic review of the current literature, this analysis seeks to determine the global incidence of BCGosis and identify critical risk factors associated with its onset.

**Methods:**

The review was conducted in accordance with the PRISMA-2020 guidelines (Preferred Reporting Items for Systematic Reviews and Meta-Analyses). The review’s objectives and scope were framed using the PICOS (Population, Intervention, Comparison, Outcomes and Study Design) framework.

**Results:**

Not surprisingly, BCGosis is most prevalent in infants with underlying genetic immunodeficiencies such as severe combined immunodeficiency (SCID) and chronic granulomatous disease (CGD). We found a high correlation between the development of BCGosis and genetic mutations affecting certain immune processes, notably those involved in NADPH oxidase function and interferon-gamma signalling. The risks of developing these mutations also correlated with the prevalence of consanguinity, a common practice in certain populations. Factors like early neonatal vaccination (often within the first week of life) and variations in BCG strains may also influence BCGosis risk.

**Conclusion:**

There is an urgent need for enhanced pre-vaccination screening for genetic and immunologic vulnerabilities in infants at hight risk for BCGosis, particularly in populations with high consanguinity rates. Alternatively, considerations should be made as to modifying existing vaccination schedules or postponing BCG immunization until immune competency can be confirmed in these high risk groups.

## Introduction

Tuberculosis (TB) remains one of the most challenging global health issues, accounting for approximately 10 million new cases and 1.4 million deaths annually, with a significant burden in developing countries (1). In response to this persistent threat, the Bacille Calmette-Guérin (BCG) vaccine has become the primary tool in preventing severe forms of childhood TB, especially tuberculous meningitis and miliary TB (2). Developed in the 1920s, BCG is the only vaccine currently available against TB and is typically administered shortly after birth in high-burden countries as part of national immunization programs (3, 4). Each year, approximately 100 million infants worldwide receive the BCG vaccine (5). The vaccine’s efficacy is well-documented, with studies showing a protective effect of up to 80% against life-threatening forms of TB in young children (6). However, its overall effectiveness in preventing pulmonary TB varies by region due to factors such as genetic diversity, environmental exposure to nontuberculous mycobacteria, and differences in BCG strains (7, 8). Despite these variations, the World Health Organization (WHO) strongly recommends BCG vaccination in TB-endemic countries, as the benefits in reducing severe pediatric TB outcomes are considered substantial (9).

### Global vaccination coverage and policy

Global Vaccination Coverage and Policy BCG vaccination is one of the most widely administered immunizations globally, integrated into childhood vaccination programs in over 150 countries (10). In countries with a high TB burden, vaccination coverage often exceeds 90%, ensuring early protection against TB-related morbidity and mortality. National policies on BCG vary, with some countries administering BCG only to high-risk groups, such as healthcare workers and individuals in close contact with TB patients, while others provide universal neonatal vaccination (11). These strategies reflect the BCG vaccine’s vital role in TB prevention, though differences in regional TB epidemiology and healthcare infrastructure influence policy decisions.

### Overview of BCGosis

Although generally safe, the BCG vaccine is associated with rare adverse events, among which BCGosis is one of the most severe (12–14). BCGosis following BCG vaccination is characterized by the spread of BCG bacteria from the injection site to multiple organs, leading to widespread granulomatous inflammation (15). Clinically, BCGosis often presents within 2–8 months postvaccination (median onset of 3.1 months) and manifests with symptoms such as persistent fever, failure to thrive, hepatosplenomegaly, lymphadenopathy, and skin lesions (16). The underlying mechanism is primarily immune-mediated, where inadequate containment of BCG leads to systemic dissemination. The most common predisposing conditions include primary immunodeficiencies and impaired cell-mediated immunity (17, 18), which are discussed in detail in the following section on the epidemiology and risk factors of BCGosis.

### Epidemiology of BCGosis post-BCG vaccination

The incidence of BCGosis following BCG vaccination is low, yet it is likely underestimated due to diagnostic limitations and variable reporting across regions. Studies estimate an incidence rate of 0.06 to 1.56 cases per million BCG vaccinations, with higher rates observed in settings where immune deficiencies are more prevalent or where diagnostic resources enable better detection (19). Primary immunodeficiencies are the most significant known risk factor for BCGosis, with children having SCID or CGD at particularly high risk (17). Other potential risk factors include undiagnosed or unclassified primary immunodeficiency disorders (18), undiagnosed HIV infection (especially in high-prevalence settings where maternal HIV screening may be incomplete) (20), malnutrition (21), and early neonatal vaccination (within the first week of life) (22). Differences in BCG strains and vaccine doses have also been explored as risk modifiers, although current evidence on these factors is inconclusive (23, 24).

### Clinical impact and management of BCGosis

BCGosis is a serious complication with varied clinical outcomes, from full recovery to severe sequelae or mortality (25). Timely diagnosis and treatment are essential for improving prognosis; however, management can be challenging. Standard treatment typically includes prolonged antituberculosis therapy, sometimes lasting months, and may require additional interventions such as interferon-gamma to support immune function (26). IFN-γ plays a key role in host defense by activating macrophages, enhancing bacterial killing, and promoting granuloma formation to contain infection, as well as stimulating Th1-mediated immune responses critical for controlling intracellular pathogens (27). While some patients respond well to treatment, others may experience persistent or recurring disease. The long-term impact of BCGosis, including quality of life and potential chronic health issues in survivors, is not well-documented, underscoring the need for research on long-term outcomes and follow-up strategies.

Despite the seriousness of BCGosis following BCG vaccination, there has been no comprehensive systematic review or meta-analysis that specifically addresses this complication in infants (28, 29). Past studies have primarily focused on general adverse events associated with the BCG vaccine, lacking the depth of meta-analytic rigor needed to compile incidence rates and risk factors across diverse populations. Gaining a thorough understanding of the epidemiology, risk factors, and clinical outcomes related to BCGosis is crucial for multiple reasons. First, reliable data on BCGosis incidence and outcomes would allow healthcare providers and policymakers to make informed decisions about BCG vaccination policies, particularly as new TB vaccines emerge. Second, identifying and quantifying risk factors can facilitate targeted efforts to minimize the risk of BCGosis, such as screening for immunodeficiencies prior to vaccination and adjusting the timing of vaccinations for infants deemed high-risk. Third, synthesizing data on clinical presentation and treatment outcomes could contribute to the development of evidence-based guidelines that improve BCGosis management. Finally, addressing current knowledge gaps in the epidemiology, risk assessment, and long-term outcomes of BCGosis would guide future research priorities and ultimately enhance patient care.

This systematic review and meta-analysis aim to address critical gaps in understanding BCGosis in infants following BCG vaccination. Specifically, it seeks to determine the global and regional incidence rates of BCGosis in newborns, with a focus on potential variations across different healthcare settings. This work also aims to identify risk factors associated with BCGosis, examining both host-related factors, such as genetic predispositions, and vaccine-specific factors, including strain and dosage. Additionally, the study will collate clinical data on the presentation, diagnostic challenges, management strategies, and outcomes of BCGosis cases to better understand the range of clinical experiences and effective treatments. An assessment of the quality and potential biases in existing studies will be conducted, highlighting areas where methodological improvements are needed. Finally, the study will explore preventive strategies that could support early identification, diagnosis and intervention within national immunization programs. By consolidating and critically analyzing the available evidence, this research aims to deepen our understanding of BCGosis following BCG vaccination in infants, thereby providing insights that could guide clinical practice, inform vaccination policies, and shape future research in neonatal immunization and TB prevention.

## Methodology

This systematic review was conducted in accordance with the PRISMA-2020 guidelines (Preferred Reporting Items for Systematic Reviews and Meta-Analyses), ensuring a structured and transparent approach throughout each phase of the research process, from literature search to data synthesis. The review’s objectives and scope were framed using the PICOS (Population, Intervention, Comparison, Outcomes, and Study Design) framework, as outlined in **Table 1** below.

**Table 1:** PICOS framework.

PICOS	Description
P (Participants)	Infants.
I (Intervention)	BCG Vaccination.
C (Comparisons)	Infants without BCGosis or those not vaccinated with BCG.
O (Outcomes)	Incidence of BCGosis, associated risk factors, and clinical outcomes (morbidity, mortality, long-term health effects).
S (Study Designs)	Human studies including randomized control studies, observational studies, cross-sectional studies, and cohort studies.

A comprehensive search strategy was developed to identify studies investigating the incidence, risk factors, and outcomes associated with BCGosis following BCG vaccination in newborns. The search was conducted across major medical databases—PubMed, Google Scholar, MedLine, Web of Science, Scopus, Embase, and ScienceDirect—chosen for their extensive coverage of clinical and medical research literature. To maximize retrieval accuracy, the search query utilized a combination of keywords and Medical Subject Headings (MeSH) terms, including: (“Newborns” OR “Neonates” OR “Infants”) AND (“BCG vaccine” OR “Bacillus Calmette-Guerin” OR “BCG vaccination”) AND (“Disseminated granulomatous disease” OR “Granulomatous disease” OR “Chronic granulomatous disease” OR “Granuloma” OR “BCG adverse effects”) AND (“Incidence” OR “Risk factors” OR “Morbidity” OR “Mortality” OR “Outcomes” OR “Clinical outcomes” OR “Long-term health effects”). Boolean operators (AND/OR) structured the search syntax to identify studies focused on the development of BCGosis post-BCG vaccination, specifically addressing the incidence, associated risk factors, and clinical outcomes in infants. This search was performed across titles, abstracts, and keywords, concluding on 03/10/2024. All references were imported into Rayyan software for processing, including duplicate removal and initial screening.

Inclusion and exclusion criteria were rigorously defined to ensure the systematic review’s focus remained on high-quality studies relevant to the research objectives. The inclusion criteria specified peer-reviewed original research articles involving newborns, neonates, and infants who received BCG vaccination as a preventive measure against tuberculosis. The primary outcomes evaluated included the incidence of BCGosis, risk factors contributing to its development, and clinical outcomes, such as morbidity, mortality, and long-term health effects. Eligible study types included case reports, case series, randomized controlled trials, observational studies, cross-sectional studies, cohort studies, and retrospective studies. Exclusion criteria applied to non-peer-reviewed literature (such as editorials, opinion pieces, and conference reports), studies related to medications, animal studies, and articles not published in English.

The screening process was systematic and thorough, beginning with a title screening followed by an abstract screening to apply the inclusion and exclusion criteria. This was followed by a detailed full-text review, where studies were further assessed for relevance and scientific rigor, focusing on those that provided specific insights into the risk factors, incidence, and clinical outcomes of BCGosis following BCG vaccination. The final selection of studies aimed to build a robust and relevant dataset, ensuring the systematic review’s comprehensiveness and relevance to the research objectives.

Data extraction was conducted using a structured Microsoft Excel form to systematically capture essential information from each study. The extraction sheet documented the author, publication year, study design, sample size, patient demographics (age, weight, gender, nationality), genetic background, family history, immune status, comorbidities, details of the BCG vaccine (strain, timing, dosage), signs and symptoms, BCGosis incidence, complications, mortality rates, long-term health effects, and the number of patients who did not develop BCGosis. This structured approach to data extraction allowed for an organized synthesis of findings on BCGosis incidence, risk factors, and clinical outcomes in infants post-BCG vaccination.

The risk of bias (ROB) in the included studies was assessed using Critical Appraisal Skills Programme (CASP) checklists tailored for cohort studies (30). The CASP checklists offer structured criteria to evaluate the methodological quality and validity of each study, ensuring a thorough examination of potential biases. The CASP cohort checklist was used, which focuses on evaluating selection bias, assessment of exposure, and outcome measurement over time. It also examines the adequacy of follow-up and controls for confounding variables, which are crucial for establishing the reliability and applicability of findings in observational research. By applying this checklist, a comprehensive and rigorous appraisal of the studies’ quality was achieved, ensuring that the findings of this systematic review were based on robust evidence with minimized risk of bias.

Data analysis of the extracted outcomes was performed on R software (version 4.4.1) using RStudio. To find the pooled proportions and confidence interval (CI), forest plots were drawn using the inverse variance method. The random effect model was used when heterogeneity (I^2^) was high (40% to 100%) while the fixed model was applied when across studies heterogeneity was less (40%). A P-value less than 0.05 was considered significant to assess the significance of the heterogeneity. We generated Doi plots for each outcome along with the Luis Furuya-Kanamori (LFK) index to evaluate the publication bias. Since our meta-analysis included fewer studies, Doi plots and LDK index were used because of their high sensitivity. An LFK index of <|1| indicates that the plot is asymmetrical. Minor symmetry is indicated by an LFK index between |1| and |2|, whereas major asymmetry is indicated by an LFK index >|2| (38).

## Results

### Study selection

The initial phase of screening for the studies identified in this systematic review involved evaluating their Titles and Abstracts to preliminarily determine relevance according to the PICOS criteria established for this review. A comprehensive search yielded 1,632 records, which were imported into Rayyan software for organized screening management. Rayyan’s automatic detection feature identified and removed 135 duplicate entries, resulting in 1,497 unique records for further evaluation.

In the first screening stage, each Title and Abstract was carefully reviewed based on predefined inclusion and exclusion criteria. This initial filter excluded 1,304 studies that did not align with the review’s primary focus—examining the incidence and risk factors for BCGosis following BCG vaccination in infants. The remaining 193 records underwent a secondary screening phase, which involved a closer examination of abstracts to ensure alignment with the study’s objective of investigating risk factors related to the incidence of BCGosis in infants. Studies that did not directly address this topic were excluded, narrowing the selection to 31 articles for a full-text review.

**Figure 1:**
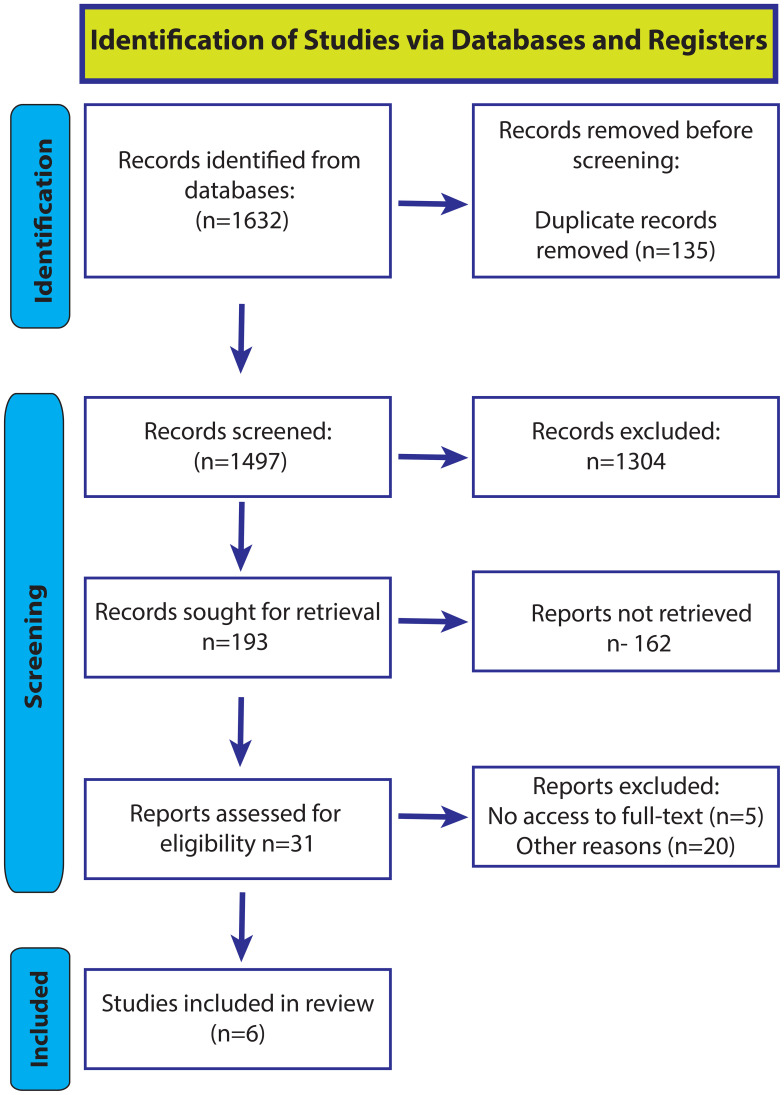
PRISMA-2020 flow chart illustrating the methodology of the study.

In the final stage, full-text articles were meticulously evaluated for adherence to the inclusion criteria. This resulted in the exclusion of 25 studies due to insufficient focus or relevant data, leaving 6 studies deemed suitable for inclusion in the systematic review. This multi-stage screening process established a methodologically rigorous foundation for understanding BCGosis following BCG vaccination in infants. A PRISMA flowchart (**Figure 1**) was developed to visually illustrate this process, providing transparency from the initial search to the final study selection (31).

### Risk of bias assessment

Based on the CASP (Critical Appraisal Skills Programme) checklist for cohort studies, the risk of bias (ROB) analysis was conducted for six studies: Reetika et al., 2020 (32); Aelami et al., 2015 (33); Li et al., 2019 (34); Trevenen et al., 1982 (35); Poudel et al., 2014 (36); and Paiman et al., 2006 (37), which is summarized in **Supplementary Table 1**. Each study was assessed across twelve questions covering aspects like cohort recruitment, measurement accuracy, confounding factors, and relevance of results. Most studies addressed a clearly focused issue and recruited their cohorts in an acceptable way, yielding low risk in these categories.

For questions about minimizing bias in exposure and outcome measurement, the studies generally performed well, with most scores marked as “2,” indicating good practices. However, in assessing confounding factors, some studies showed a moderate risk. For instance, Reetika et al. (32), Aelami et al. (33), and Trevenen et al. (35) scored “1” in identifying important confounding factors, while Poudel et al., 2014– (36) received a score of “0” in accounting for confounding factors, suggesting potential limitations in their designs.

Regarding follow-up adequacy, most studies scored reasonably well, though Poudel et al., 2014– (36) again scored lower, indicating a higher risk of bias due to incomplete follow-up. On applicability to the local population, alignment with other evidence, and implications for practice, the studies generally scored well, suggesting that their findings could be useful for broader application. The overall risk scores varied, with Poudel et al., 2014 (36), having the highest risk (18 points), while Trevenen et al., 1982 (35), had the lowest (26 points). These scores suggest moderate to high quality for most studies, with some areas of potential bias, particularly in confounding factors and follow-up completeness.

### Study characteristics

The review included six studies investigating the incidence, risk factors, and outcomes of BCGosis following BCG vaccination in infants. Study designs varied, encompassing retrospective cohort studies [Paiman et al., 2006 (37); Aelami et al., 2015 (33); Reetika et al., 2020 (32)], prospective cohort studies (Li et al., 2016 (34)), and individual case reports [Trevenen et al., 1982 (35); Poudel et al., 2014 (36)]. Across these studies, a total of 256 patients from different ethnic backgrounds and geographic locations were examined, allowing insights into disease progression and outcomes in varied healthcare and cultural settings. Key data extracted from each study are summarized in **Supplementary Table 2**.

### Population and demographics

The studies collectively focused on pediatric populations, with ages ranging from less than a month [Reetika et al., 2020 (30)) to over three years (Li et al., 2016 (32)]. Studies were conducted in regions with ethnically diverse populations, including Canadian Inuit, Indian, Iranian, Nepalese, and Chinese participants, thereby providing a broad perspective on the occurrence and clinical presentation of BCGosis. Notably, the Paiman et al., 2006– (35) and Reetika et al., 2020– (30) studies indicated a high prevalence of consanguineous families among affected infants, often associated with genetic immunodeficiencies.

Several studies emphasized the correlation between genetic factors and disease development. For example, Li et al., 2016– (32) reported X-linked recessive mutations (for example, CYBB and CYBA) in Chinese infants, while Reetika et al., 2020– (30) identified specific mutations such as IL2RG, JAK3, ADA, and defects in IFN-γ signaling pathways (such as IFNγR1/2, STAT1). These genetic vulnerabilities, often exacerbated by regional consanguinity rates, were significant contributors to the incidence of BCGosis, suggesting that genetic predispositions may play a central role in this population.

### Vaccination and follow-up

BCG vaccination protocols, including strains, doses, and timing, varied among studies. Most studies reported early neonatal vaccination, with administration within the first 24 hours of life. The Pasteur strain was used in several Iranian cases (Paiman et al., 2006 (35); Aelami et al., 2015 (31)), while the D2PB302 strain was common in Chinese cohorts [Li et al., 2016 (32)]. Trevenen et al., 1982– (33) and Poudel et al., 2014– (34) did not specify BCG strain but provided standard infant dosing data.

Follow-up durations ranged from immediate post-vaccination observations to longer-term surveillance. For instance, Li et al., 2016– (32) followed patients for a mean of 35.2 months, capturing delayed onset of BCGosis in immunocompromised infants. Conversely, the study by Trevenen et al., 1982 (33), based on autopsy data, provided only case-specific findings without extended follow-up. This variability in follow-up durations highlights differences in surveillance practices across regions and the importance of extended monitoring in populations at high risk of BCGosis complications.

### Inclusion criteria

Inclusion criteria across studies focused on infants with a confirmed history of BCG vaccination and clinical or histopathologic evidence of disseminated mycobacterial infection. Paiman et al., 2006– (35) required a positive BCG inoculation history, systemic symptoms compatible with mycobacterial disease (such as, fever, lymphadenopathy), and histological evidence of BCG infection in at least two non-vaccination sites. Aelami et al., 2015– (31) and Li et al., 2016– (32) specified similar criteria, including the presence of acid-fast bacilli in multiple organs beyond the initial vaccination site. In addition, many studies included a requirement for testing immune status; for example, Reetika et al., 2020– (30) and Aelami et al., 2015– (31) tested for severe immunodeficiencies, finding a high prevalence of SCID, CGD, and other inborn errors of immunity.

The comprehensive examination of study demographics, genetic factors, vaccination protocols, and inclusion criteria provides a foundation for understanding BCGosis disease progression and risk factors. These findings underscore the role of early genetic screening and prolonged follow-up in managing BCGosis risk in vulnerable infant populations across diverse global settings.

### Incidence of BCGosis

The incidence of BCGosis varied across studies, but certain patterns were evident, particularly in cases with genetic vulnerabilities. Paiman et al., 2006– (35) investigated 17 infants in Iran, who developed BCGosis after BCG vaccination. These cases showed systemic signs, including multi-organ involvement, fever, weight loss, lymphadenopathy, hepatomegaly, splenomegaly, and pneumonia. Only infants with confirmed evidence of BCG infection in at least two anatomical sites beyond the vaccination region were included, while 13 infants were excluded underscoring a rigorous approach that revealed a significant incidence of BCGosis in susceptible groups.

In the study by Reetika et al., 2020 (30), 6,925 BCG-vaccinated infants from India were screened, and 90 cases presented with BCG-related complications. Of these, 43 cases were classified as probable BCGosis, while 47 were possible cases. Many of these infants showed extensive granulomatous disease in organs beyond the initial vaccination site, with lymphadenitis, microabscesses, and hepatosplenomegaly as common symptoms. Among this group, infants with genetic defects—particularly those with IFNγR1/2 and STAT1 deficiencies—accounted for a high proportion of disseminated BCG complications, demonstrating a considerable incidence of BCGosis in genetically predisposed infants.

23 out of 78 patients included in the study conducted by Li et al., 2016– (32) developed BCGosis. Initial symptoms of these infants post BCG vaccination are fever, multiple lymph node enlargement, cough, ascites, and diarrhea. Later, these infants developed dissemination to pulmonary, multiple systemic lymph nodes, liver, pericardium, soft tissues, nervous system, penis, nose, ears and skin. Some of them also developed splenomegaly, hydronephrosis, and bone lesions.

#### Pooled effects:

**Figure 2:**
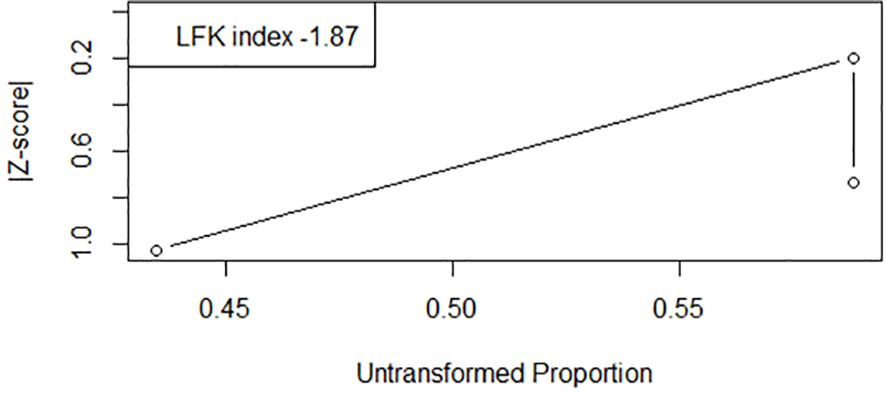
Forest plot illustrating the incidence of BCGosis.

**Figure 3:**
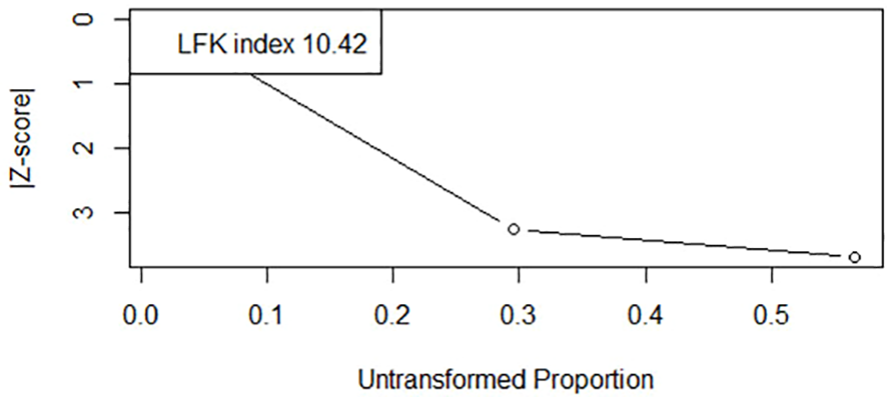
DOI plot illustrating the incidence of BCGosis.

The pooled incidence rate of BCGosis following BCG vaccination was 0.28(95% CI: 0.00 to 0.59) with high heterogeneity (97%, Chi (2) = 67.20, p< 0.01) across studies indicating significant variability among studies potentially due to population characteristics (**Figure 2**). The LFK index calculated was 10.42 showing major asymmetry and substantial publication bias in the Doi plot as shown in **Figure 3**.

### Risk Factors for BCGosis

The risk factors for BCGosis following BCG vaccination fell into several main categories, including genetic mutations, immune deficiencies, family history and consanguinity, and general health factors.

#### Genetic mutations and immune deficiencies

BCGosis is predominantly associated with genetic mutations that lead to significant immunodeficiencies. Mutations affecting the NADPH oxidase complex, including CYBB, CYBA, NCF1, and NCF2, compromise neutrophil function and the production of reactive oxygen species, predisposing patients to chronic granulomatous disease (CGD) and heightened susceptibility to BCGosis (Li et al., 2016; Reetika et al., 2020) (32, 34). Similarly, defects in interferon gamma signaling, such as mutations in IFNγR1, IFNγR2, and STAT1, impair the host’s ability to control mycobacterial infections and are frequently observed in affected individuals (Reetika et al., 2020) (32).

Severe combined immunodeficiency (SCID), resulting from mutations in genes such as IL2RG, JAK3, and ZAP70, leads to profound T and B cell deficiencies, rendering infants incapable of containing the BCG vaccine, which disseminates rapidly to multiple organs (Reetika et al., 2020) (32). In addition, other rare inborn errors of immunity, including unidentified cell-mediated defects, further disrupt macrophage and neutrophil function, increasing vulnerability to disseminated mycobacterial infection (Aelami et al., 2015; Li et al., 2016) (33, 34). For instance, Aelami et al., 2015– (33) reported that 17 out of 34 BCGosis cases had various immune deficiencies, with unidentified cell-mediated immune defects or CGD commonly found. Collectively, these genetic and immunological abnormalities constitute the primary risk factors for BCGosis, underscoring the central role of compromised innate and adaptive immunity in its pathogenesis.

These immunodeficiencies also render infants more susceptible to additional infections, which may further exacerbate disease severity. Reetika et al., 2020– (32) reported cases of BCGosis occurring alongside infections such as tuberculosis or Epstein-Barr virus, while Li et al., 2016– (34) identified 14 patients with concurrent infections. Such findings indicate that impaired immune function not only predisposes infants to BCGosis but also diminishes the capacity to control other pathogens, amplifying the overall risk and severity of disease.

#### Family history and consanguinity

Family History of BCG Complications: Paiman et al., 2006– (37) observed that 41.17% of infants with BCGosis had a family history of BCG complications, suggesting inherited susceptibility factors. Additionally, Li et al., 2016– (34) reported families with previous infant mortality or siblings with unexplained infections, suggesting that family history was an indicator of risk, likely due to inherited immune deficiencies. Consanguinity: Consanguinity was a prominent factor in BCGosis risk. In Paiman et al., 2006 (37), 82.35% of affected families reported consanguineous marriages, while Li et al., 2016– (33) and Reetika et al., 2020– (32) noted similar trends. This finding indicates that closely related parents increase the likelihood of genetic mutations associated with immune dysfunction, making BCGosis more likely.

#### General health factors and co-morbidities

Trevenen et al., 1982– (35) noted that two cases of BCGosis might have been exacerbated by malnutrition, which can temporarily impair immune function. In these cases, malnutrition likely weakened the infants’ immune systems, increasing their susceptibility to BCG dissemination.

### Mortality and morbidity

Across all studies, mortality rates were alarmingly high among infants with immune deficiencies who developed BCGosis after BCG vaccination. Paiman et al., 2006– (35) reported a mortality rate of 58.8%, with infections spreading rapidly in immunocompromised infants. Reetika et al., 2020– (30) observed a 100% mortality rate in infants with severe combined immunodeficiency (SCID), while long-term survivors dealt with severe complications, including chronic lymphadenitis, infections, and abscesses. Similarly, Li et al., 2016– (32) found a 44% mortality rate, noting that surviving infants suffered from ongoing inflammation and multi-organ involvement. Aelami et al., 2015– (31) also reported a 58.8% mortality rate among their 34 cases, with severe infections leading to fatal outcomes. These findings indicate that infants with underlying immune deficiencies, particularly those with SCID and chronic granulomatous disease (CGD), face significantly increased risks of mortality and morbidity. Survivors often experience persistent, multi-organ complications that demand prolonged medical intervention and care.

#### Pooled mortality rates

**Figure 4:**
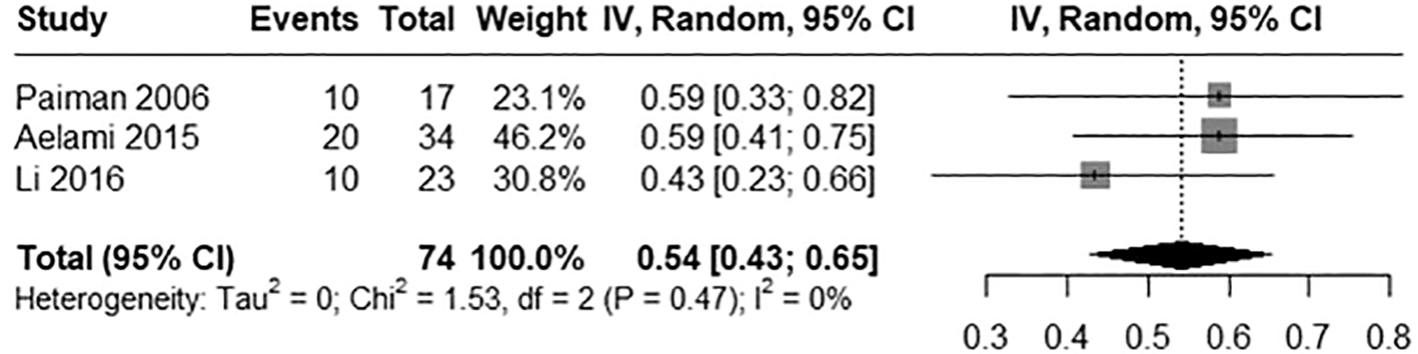
Forest plot illustrating the mortality rate of infants with BCGosis.

**Figure 5:**
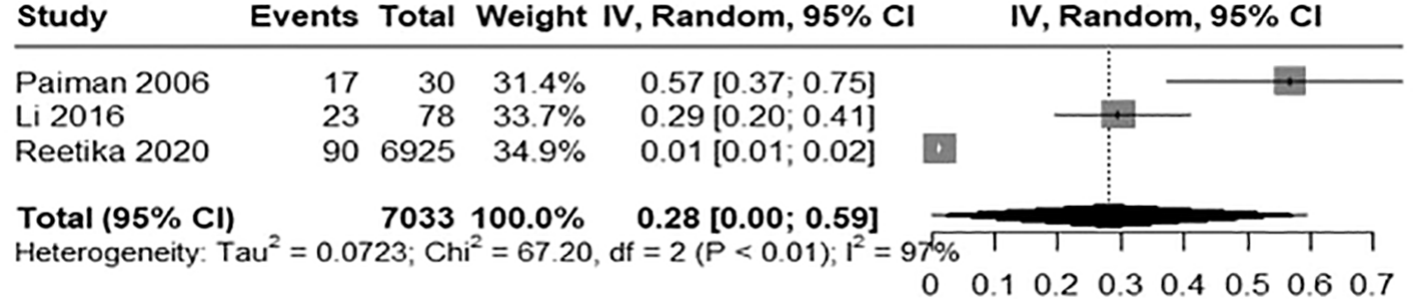
DOI plot illustrating the mortality rate of infants with BCGosis.

The pooled mortality rate was calculated as 0.54 (95% CI: 0.43 to 0.65) indicating a mortality rate of 54% among patients with BCGosis following BCG vaccine (**Figure 4**). No significant heterogeneity was detected across included studies (I2 = 0%, Chi2 = 1.53, p= 0.47) increasing the evidence of the robustness of results. An LFK index of -1.87 was calculated indicating minor asymmetry among the studies and hence less publication bias. The Doi plot is shown in **Figure 5**.

## Discussion

The findings from this systematic review and meta-analysis offer crucial insights into the incidence, risk factors, and outcomes associated with BCGosis following BCG vaccination in infants. By systematically reviewing data from six studies spanning diverse geographic regions and population demographics, this review underscores the significance of genetic and immunologic vulnerabilities in the development of BCGosis and highlights the clinical complexity of managing this condition in high-risk infants.

### Summary of key findings

The primary risk factors identified across studies were genetic mutations, immune deficiencies, family history of BCG complications, and consanguinity. Mutations in genes associated with immune pathways—particularly those involved in NADPH oxidase and interferon-gamma receptor signaling—were consistently linked to increased susceptibility to BCGosis. This association was most evident in infants with severe combined immunodeficiency (SCID) or chronic granulomatous disease (CGD), for whom mortality rates were particularly high. Family history and consanguinity, common in several studied populations, emerged as additional risk factors, likely due to the inheritance of genetic mutations affecting immune function.

### Genetic vulnerabilities and immune deficiencies

The role of genetic predispositions in BCGosis development is substantial. Specific mutations, such as those in the CYBB, IL2RG, and STAT1 genes, impair the body’s ability to mount effective immune responses, particularly against mycobacterial pathogens like BCG. High consanguinity rates in some regions further complicate this genetic predisposition, increasing the prevalence of inherited immune deficiencies. The clinical implications of these findings underscore the importance of genetic screening in high-risk populations, particularly in regions with high rates of consanguineous marriages. Early identification of infants with genetic mutations linked to immune dysfunction could inform preemptive strategies, such as alternative vaccination schedules or targeted clinical monitoring, to mitigate the risk of BCGosis.

### Incidence and population impact

Incidence rates of BCGosis varied, but infants with underlying immunodeficiencies consistently showed a higher risk of developing severe complications. Paiman et al., 2006–37 and Reetika et al., 2020– (32) highlighted the prevalence of consanguinity in affected infants, suggesting that genetic predispositions significantly influence disease incidence in certain populations. The variability in study designs, with some studies focusing on retrospective data while others followed cases prospectively, contributes to discrepancies in incidence rates and complicates comparisons across regions. However, the high mortality rates observed, especially in cases with severe immunodeficiencies, emphasize that BCGosis poses a considerable health burden on vulnerable populations.

### Implications for clinical practice

The findings emphasize the necessity of tailored healthcare approaches for infants at high risk of BCGosis, particularly those with known or suspected immunodeficiencies. Consistent with recommendations in the reviewed studies, routine immunologic and genetic screenings for infants with a family history of immune disorders or high-risk genetic backgrounds could improve early detection and intervention for BCGosis. Extended follow-up protocols are also crucial, given that some BCGosis cases may develop months or years post-vaccination. Implementing standardized follow-up protocols that combine routine clinical evaluations with immunological and microbiological monitoring would allow healthcare providers to identify complications at an earlier stage, initiate timely interventions, and thereby reduce mortality while also addressing chronic sequelae such as recurrent infections and lymphadenitis.

Additionally, variations in BCG vaccination strains, doses, and timing observed across studies suggest a potential area for further research to determine if certain BCG strains may pose a greater risk to infants with immune vulnerabilities. Evidence indicates that BCG sub-strains are not immunologically identical: some, such as the Pasteur and Danish strains, may induce stronger immune responses but have also been associated with higher rates of adverse events, including BCGosis infection. In contrast, strains like Tokyo or Russian appear to generate more moderate immune activation with fewer reported complications (Ritz et al., 2008; Dagg et al., 2014) (23, 24). These strain-specific differences may partly explain the heterogeneity in outcomes reported across regions and highlight the importance of considering sub-strain type when interpreting complication rates. Future comparative studies that directly evaluate the safety and immunogenicity of individual sub-strains in neonates with suspected immunodeficiencies would provide critical evidence to inform global vaccination policies.

In line with this, universal neonatal screening for Severe Combined Immunodeficiency (SCID) should be considered as a preventive strategy. Early detection allows for timely interventions, such as hematopoietic stem cell transplantation, and prevents inadvertent BCG vaccination in affected infants. Several high-income countries have already integrated SCID screening into their newborn screening panels using T-cell receptor excision circle (TREC) assays, demonstrating both feasibility and cost-effectiveness. Implementing such programs more broadly, particularly in countries with high consanguinity rates and high reliance on neonatal BCG vaccination, could substantially reduce the incidence of BCGosis. Beyond safeguarding infants with SCID, these programs also serve as a gateway to identifying other inborn errors of immunity, thereby improving overall pediatric outcomes.

### Limitations and future research directions

This meta-analysis has several limitations that must be acknowledged. First, the heterogeneity in study designs and inclusion criteria across the reviewed studies presents challenges in synthesizing findings. Additionally, variability in follow-up durations and data reporting practices complicates a direct comparison of outcomes across different settings. There is also a need for more data on the long-term health impacts of BCGosis on surviving infants, as few studies reported detailed information on morbidity beyond the immediate post-vaccination period. Future research should focus on larger, multicenter cohort studies with standardized methodologies to enhance the reliability and generalizability of findings. Expanding genetic research on immune pathways could further elucidate specific mechanisms driving BCGosis development, while intervention studies might assess the effectiveness of alternative vaccination strategies in high-risk populations.

## Conclusion

This systematic review and meta-analysis provides critical evidence that genetic and immunologic factors significantly contribute to the incidence and severity of BCGosis following BCG vaccination. These findings highlight the importance of targeted healthcare strategies for infants with identified immune vulnerabilities, including genetic screening and extended follow-up. By addressing the risk factors associated with BCGosis, healthcare providers and policymakers can better protect at-risk infants and reduce the substantial health burden posed by this serious post-vaccination complication.

## Data Availability

The original contributions presented in the study are included in the article/**Supplementary Material**. Further inquiries can be directed to the corresponding author/s.
