# Supplementary material for: Incidence, risk factors and outcomes of BCGosis following BCG vaccination in infants: a systematic review and meta-analyses
Source: Front Immunol. 2025 Dec 11;16:1615039. doi: 10.3389/fimmu.2025.1615039 (PMC12738926; doi:10.3389/fimmu.2025.1615039)
Supplement: Supplementary file 1 [file Table1.docx]

*Supplementary Table 1: Risk of Bias Analysis Using the Critical Appraisal Skills Programme (CASP) tool – for cohort study*

| **Study** | **Question 1** | **Question 2** | **Is it worth continuing?** | **Question 3** | **Question 4** | **Question 5a** | **Question 5b** | **Question 6a** | **Question 6b** | **Question 7** | **Question 8** | **Question 9** | **Question 10** | **Question 11** | **Question 12** | **Overall risk** |
| --- | --- | --- | --- | --- | --- | --- | --- | --- | --- | --- | --- | --- | --- | --- | --- | --- |
| Reetika et al., 2021 ^32^ | 2 | 2 | yes | 2 | 2 | 1 | 2 | 1 | 1 | 1 | 1 | 2 | 2 | 1 | 1 | 21 |
| Aelami et al., 2015 ^33^ | 2 | 2 | yes | 2 | 2 | 1 | 2 | 1 | 1 | 1 | 2 | 2 | 2 | 2 | 2 | 24 |
| Li et al., 2019 ^34^ | 2 | 2 | yes | 2 | 2 | 2 | 2 | 2 | 2 | 1 | 1 | 2 | 1 | 2 | 1 | 24 |
| Trevenen et al., 1982 ^35^ | 2 | 2 | yes | 2 | 2 | 1 | 1 | 2 | 2 | 2 | 2 | 2 | 2 | 2 | 2 | 26 |
| Poudel et al., 2014 ^36^ | 2 | 2 | yes | 2 | 2 | 1 | 0 | 0 | 1 | 1 | 1 | 2 | 1 | 2 | 1 | 18 |
| Paiman et al., 2006 ^37^ | 2 | 2 | yes | 2 | 2 | 1 | 2 | 1 | 1 | 1 | 1 | 2 | 2 | 2 | 1 | 22 |

Question 1: Did the study address a clearly focused issue?

Question 2: Was the cohort recruited in an acceptable way?

Question 3: Was the exposure accurately measured to minimize bias?

Question 4: Was the outcome accurately measured to minimize bias?

Question 5a: Have the authors identified all important confounding factors?

Question 5b: Have they taken account of the confounding factors in the design and/or analysis?

Question 6a: Was the follow up of subjects complete enough?

Question 6b: Was the follow up of subjects long enough?

Question 7: What are the results of this study?

Question 8: How precise are the results?

Question 9: Do you believe the results?

Question 10: Can the results be applied to the local population?

Question 11: Do the results of this study fit with other available evidence?

Question 12: What are the implications of this study for practice?
