# Supplementary material for: Incidence, risk factors and outcomes of BCGosis following BCG vaccination in infants: a systematic review and meta-analyses
Source: Front Immunol. 2025 Dec 11;16:1615039. doi: 10.3389/fimmu.2025.1615039 (PMC12738926; doi:10.3389/fimmu.2025.1615039)
Supplement: Supplementary file 2 [file Table2.docx]

*Supplementary Table 2: Summary and Key Findings of Included Studies*

| **Study** | **Country** | **Study Design** | **Sample Size** | **Age Range** | **Patient Demographics** | **BCG Strain and Dose** | **Follow-Up Duration** | **Key Findings** |
| --- | --- | --- | --- | --- | --- | --- | --- | --- |
| Trevenen et al., 1982 ^35^ | Canada | Retrospective Study | 36 | 1 - 12 months | Canadian Indian, Inuit, and Caucasian infants | Mycobacterium bovis, BCG type; 0.05 ml |  | 26 infants developed tuberculoid granulomas; malnutrition suspected to have exacerbated dissemination; deaths due to unrelated causes |
| Paiman et al., 2006 ^37^ | Iran | Retrospective Study | 17 | 3 - 36 months | 47% male, 53% female; consanguineous family history in 82.35% | Pasteur strain; standard dose | Case specific | 58.8% mortality; systemic symptoms included fever, lymphadenopathy, and hepatosplenomegaly; SCID and CGD prevalent in immunocompromised cases |
| Poudel et al., 2014 ^35^ | Nepal | Case Report | 1 | 7 months | Nepalese infant | Mycobacterium bovis-derived; standard dose | Case specific | Disseminated BCG infection with axillary lymphadenopathy, hepatosplenomegaly, and weight loss; infant discharged but with possible persistent issues |
| Aelami et al., 2015 ^33^ | Iran | Retrospective study | 34 | 1 - 6 months | 41% male, 59% female; SCID identified in 12 cases | Pasteur 1173 M. bovis BCG substrain; standard dose | Routine vaccination | 58.8% mortality; systemic involvement with fever, lymphadenopathy, and hepatosplenomegaly; anemia, leukopenia observed in severe cases |
| Li et al., 2019 ^34^ | China | Prsopective cohort | 78 | 2.9–123.7 months | Chinese, mostly from the southeast region; CYBB mutation present | D2PB302 strain; national standard dose | Mean 35.2 months (6.2–102 months) | 44% mortality; disseminated infection spread to lungs, liver, lymph nodes, GI tract, and other organs; common X-linked mutations, such as, CYBB |
| Reetika et al., 2020 ^31^ | India | Retrospective study | 90 | <1 month – 12+ months   \|  \| \| --- \|  \|  \| \| --- \| | 68.9% male, 31% female; genetic immunodeficiencies in 52 infants | Various strains, not specified |  | High prevalence of SCID and CGD; complications included lymphadenitis, hepatosplenomegaly, pneumonia; mortality 100% in SCID cases |
